# Relating proprioceptive embodiment to body dissatisfaction in anorexia and bulimia patients: effect of visual body images

**DOI:** 10.1007/s00406-025-01982-5

**Published:** 2025-03-12

**Authors:** Virginia Guillén, Pedro Muñoz, Jaime Zubero-Linaza, Zigor Aira, Itsaso Buesa

**Affiliations:** 1 Department of Neurosciences, Faculty of Medicine and Nursing, University of the Basque Country UPV/EHU, Barrio Sarriena s/n, 48940 Leioa, Bizkaia Spain; 2 Biocruces-Bizkaia Health Research Institute, 48903 Barakaldo, Bizkaia Spain; 3 Bizkaia Mental Health Network (RSMB), Basque Health Service-Osakidetza, 48010 Bilbao, Spain; 4 Department of Nursing I, Faculty of Medicine and Nursing, University of the Basque Country UPV/EHU, 48940 Leioa, Bizkaia Spain; 5 Basic and Applied Psychobiology, Faculty of Psychology, Universidad Internacional de La Rioja UNIR, 26006 Logroño, La Rioja Spain

**Keywords:** Proprioception, Body image, Eating disorders, Maladaptive body schema, Rubber hand illusion

## Abstract

Eating disorders (ED) are associated with a maladaptive body schema and several cognitive biases. This pilot study aimed to investigate the effect of visual stimulation by body images on maladaptive body schema and body dissatisfaction in patients with ED. The rubber hand illusion (RHI) was applied to a sample of 33 women with anorexia or bulimia nervosa and 27 control subjects. The RHI was administered in a novel way using a standard-sized hand that had been distorted in appearance (perceived as unsatisfactory), and it was used before and after an ad hoc priming effect (exposure to thin-body media images). In accordance with the maladaptive body schema, ED patients exhibited higher scores on the Body Shape and Body Perception Questionnaires (with a positive correlation between the scores) and there was a significant increase in scores for all items in the location-proprioception and agency domains. However, before the priming effect, the ED sample showed significantly lower scores on all proprioceptive drift items during the distorted RHI condition and the regression analysis demonstrated a significant association between reduced proprioceptive drift (recording a similar embodiment index to healthy subjects) and improved body dissatisfaction. Following the priming effect, the proprioceptive drift embodiment index increased, and no ANOVA interaction was recorded. The maladaptive body schema in patients with bulimia or anorexia nervosa is characterised by both distorted proprioception and high interoceptive awareness. The visual body images that are perceived as unsatisfactory play a role in preserving proprioception and consequently in reducing body dissatisfaction. Conversely, the exposure effect of thin-body ideal images is involved in the maladaptive body schema.

## Introduction

Eating disorders (EDs) are disabling, deadly, and costly mental disorders that considerably impair physical health and disrupt psychosocial functioning, with body dissatisfaction being a central psychopathological feature [1–3]. Cognitive theories of body dissatisfaction suggest that schemas related to appearance, shape, and weight influence the processing of body image [4, 5]. Consequently, the body schema may be accompanied by biases, which result in a selective focus on body image information. It is certain that the attentional biases to the distorting visual signal play an important role in body dissatisfaction in eating pathology, as evidenced by numerous studies [4–10]. However, it can be postulated that anorexia and bulimia nervosa represent an embodiment disorder, rather than being merely associated with distorting visual patterns. This encompasses a number of different sensory experiences, including affective touch, haptic perception, interoception, nociception, proprioception, and tactile perception, as well as impairment in multisensory body integration [11–14]. Therefore, the maladaptive body schema is proposed to be the core cognitive component of ED, resulting in the selective processing of information related to distorting visual stimuli [15]. The body schema is regarded as a dynamic entity, highly dependent on ongoing proprioceptive input, which reflects the body's position and movement in space. The body image is conceptualised as the cognitive representation of the body, with a clear modulation between both [16–18]. In accordance with this, the lack of the ability to adequately update body representations in ED patients alters body perception through a negative cognitive bias toward one’s body representations, although no patterns of distortions in sensorimotor perception have been reported in anorexia or bulimia nervosa patients [19–24]. In this context, it has been postulated that the role of proprioception and body awareness may contribute to the distorted perceptual representation of body size in anorexia nervosa [16]. Recent studies, along with some neuroanatomical evidence [25–27], suggest that there may be alterations in proprioceptive perception related to body image representation [28]. Nevertheless, there is a paucity of research examining the role of proprioceptive integration in the development of a maladaptive body schema in patients with eating disorders.

The main objective of this study is to examine the visuo-tactile-proprioceptive integration in the context of maladaptive schema hypothesis using the Rubber Hand Illusion (RHI) paradigm, which allows the visual stimulus to be experimentally manipulated during the experience and provides a useful quantitative measure of embodiment; it refers to the sense of one's own body and the plasticity of the body schema [29–33]. The RHI is typically assessed using behavioural measures, which include a drift of the perceived position of the subject’s hand toward the rubber hand. This phenomenon, known as proprioceptive drift, is useful for evaluating the intensity of proprioceptive embodiment. Additionally, perceived agency (motor control) over the rubber hand is also assessed. A number of studies have demonstrated that individuals with eating disorders exhibit distortions in their body representation and are more susceptible to illusions than controls [34, 35]. Nevertheless, contradictory findings have been reported in different experimental paradigms [36], and a direct correlation between body dissatisfaction and distorted body representation in ED patients has not yet been confirmed. However, some data suggest that exposure to images of thin-body media may trigger body dissatisfaction and a maladaptive body schema through social comparison and self-discrepancy [10]. This effect is particularly pronounced in individuals who are susceptible to a thinness schema and exhibit high levels of body dissatisfaction [9, 37, 38].

The main hypothesis of the present study is that exposure to a distorting visual stimulus may trigger impairment in visuo-tactile-proprioceptive integration, with a correlation with one’s own perceived dissatisfaction. Specifically, it is proposed that women with bulimia or anorexia nervosa (BN-AN) would show a relationship between distorted body representation (mainly proprioceptive distortion) and distorting visual patterns, leading to a maladaptive body schema with high body dissatisfaction. This pilot study compares a sample of women with bulimia or anorexia nervosa (BN-AN) with a group of healthy women.

To illustrate this, a modified RHI was conducted with visual body image distortion compared to the standard procedure. Proprioceptive drift and the agency embodiment index [31] were recorded. In particular, the modified RHI was conducted with an aesthetically incongruent but normally sized rubber hand (rated as unsatisfactory). To test for cognitive bias in body representation, the study participant was then exposed to visual media images of thin bodies, and the RHI was performed again. To ascertain whether there were any changes in body dissatisfaction as a result of body representation plasticity, ratings were collected at different experimental times: at the beginning of the procedure (baseline score), at the end of the RHI before the priming, and at the end of the second RHI after priming exposure. Finally, to explore if the bodily perception of internal perception was associated with a maladaptive body schema, we recorded self-rated body awareness using the Body Perception Questionnaire (BPQ) and the Body Shape Questionnaire (BSQ).

## Materials and methods

### Study participants and organizational design

The healthy control group consisted of female students recruited at the University of the Basque Country UPV/EHU. Data collection commenced on November 15, 2021, and concluded on January 15, 2022. The experimental procedure was conducted in the university laboratory by researchers I.B. and V.G. The presence of an eating disorder was excluded from the healthy controls by administering the Eating Disorder Examination EDE [39] and using the SCID-P structured interview [40]. Female patients were recruited from an eating disorders care program at the Ortuella Mental Health Centre, part of the Bizkaia Mental Health Network (RSMB)-Osakidetza. The recruitment period commenced on April 27, 2022, and concluded on July 29, 2022. Patients were diagnosed using the Eating Disorder Examination (EDE) [39], according to DSM-IV TR [41] and using the SCID-P structured interview [40]. A psychiatrist and a psychologist at a 90-min clinical interview made the evaluations. The psychiatrist and the psychologist in all cases evaluated the same content. Sociodemographic variables (gender, age) and clinical variables (previous hospitalization, psychiatric comorbidity, psychopharmacological treatment and other comorbidities) were recorded. The inclusion criteria were patients diagnosed with eating disorder with anorexia nervosa (AN) or bulimia nervosa (BN) with more than 6 months of disease progression and who agreed to participate. In the patient sample, four individuals exhibited symptoms of an emotional instability disorder (BPD), 301.83.DSM-IV TR, while two patients demonstrated symptoms of a moderate major depressive disorder, 296.22.DSM-IV TR. One patient also exhibited symptoms of agoraphobia, 300.22.DSM-IV TR. The pharmacotherapy utilized for the treatment of depressive disorders included desvenlafaxine and fluoxetine.The pharmacology used to treat anxious pathology was lorazepam and diazepam. The dosage was adjusted according to the patient's response and clinical situation. The treatment of bulimic pathology involved the administration of high doses of fluoxetine, with two patients also receiving zonisamide. This was conducted in conjunction with individual or group psychotherapy, according to the specific needs of the patient. We excluded from the study patients who were regular drug users, who had severe malnutrition, who had psychotic pathology or intellectual disability and comorbid pathologies that could interfere with the assessment of body image distortion.

The Ethical Review Board of the University of the Basque Country approved the study protocol for healthy participants (CEISH 142/2021) on September 23, 2021, and the C.E.I. OSI Ezkerraldea Enkanterri Cruces Hospital Universitario de Cruces (CEI E22/24) for patients on April 26, 2022. Written informed consent was obtained from all participants before participation. None of the participants had previously been exposed to the rubber hand illusion.

### Measures

The Body Shape Questionnaire (BSQ) [42] is a 34-item self-report measure for exploring self-perception of body image and specifically identifies the presence of dissatisfaction with body image related to eating disorders. It measures a respondent's concern about body shape and the experience of “feeling fat” over the past four weeks (e.g., “Over the past four weeks, have you felt excessively large and rounded?”) [42]. Each question is assessed on a 6-point Likert scale that ranges from “never” to “always”. Higher total scores reveal greater levels of body dissatisfaction [42]. The Spanish version of the BSQ [43, 44] was used to assess body dissatisfaction on five subscales; (i) weight concern in connection with eating, (ii) unsightly aspects of obesity, (iii) general body dissatisfaction, (iv) lower body dissatisfaction, and (iv) use of vomiting or laxatives to reduce body dissatisfaction [42].

The Symptom Checklist 90 Revised (SCL-90-R) [45] is a 90-item self-report screening instrument used to assess current psychological pathology. The SCL-90-R has three global distress indexes; I. Global Severity Index (GSI), II. Positive Symptom Distress Index (PSDI) and III. Positive symptom total (PST), and nine subscales: somatization, obsessive–compulsive, interpersonal sensitivity, depression, anxiety, hostility, phobic anxiety, paranoid ideation, and psychoticism. The Spanish version of the SCL-90-R was used [46]. Participants rate items on a 5–5-point Likert scale ranging from 1 (not at all) to 5 (extremely). Higher scores indicated greater psychopathology.

The Body Perception Questionnaire [47] was used to assess self-rated body awareness. This questionnaire uses a 5-point scoring scale from “no awareness at all” to “permanent awareness” to quantify body perception and interoceptive awareness on four subscales (body perception awareness, stress response, autonomic nervous system reactivity, and stress style) [47].

The intensity of body dissatisfaction was measured using a visual analogue scale (VAS), which ranged from 0 to 10 [48]. In the present study, three visual analogue scales (VAS whole body, VAS own hand, and VAS rubber hand) were created. The VAS-whole body was used to assess general dissatisfaction in order not to focus attention on a specific body part, the VAS-hand itself was used to measure a specific body part directly involved in the illusion, and the VAS-rubber hand was used to assess the level of dissatisfaction due to its distorting and incongruent appearance about one's body image.

**Fig. 1:**
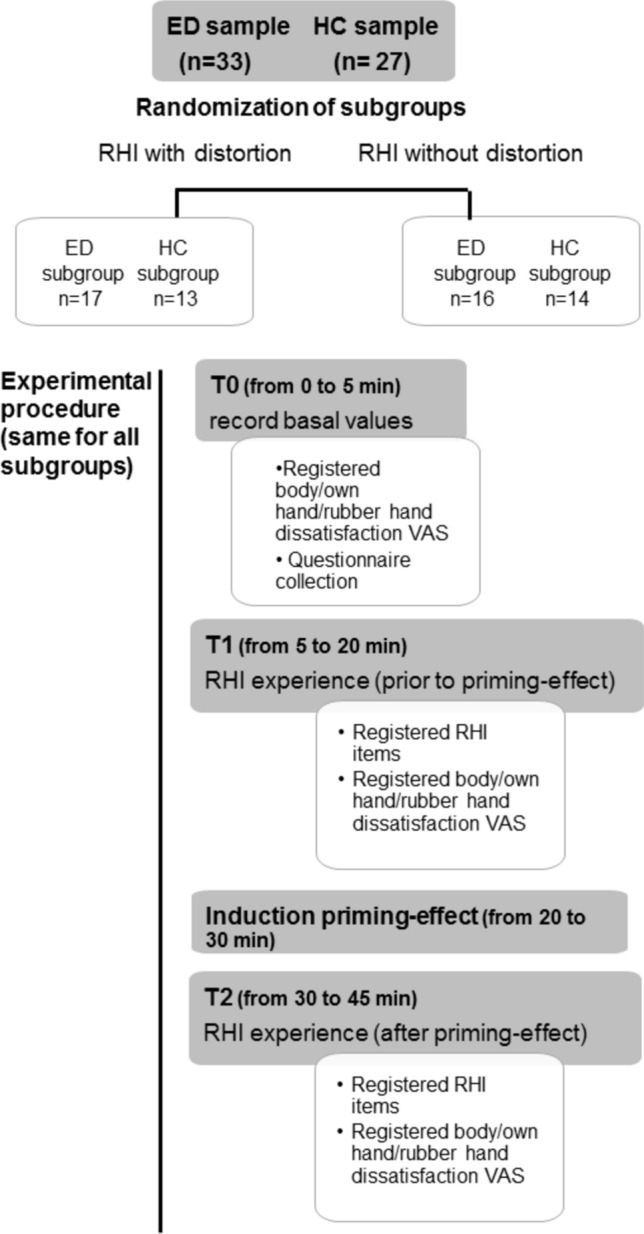
Experimental timing. The experimental procedure takes about 45 min. In each experimental sample (eating disorder group or healthy control group), two subgroups were randomly generated: the subgroup that performed RHI with a rubber hand with distorting aesthetics and the subgroup that performed RHI with a standard hand. All participants performed two RHIs (the first without priming and the second with priming). Dissatisfaction was assessed at three time points (T0 as the baseline, T1 after the first RHI, and T2 after the second RHI)

Participants were asked to rate their level of dissatisfaction in situ by drawing a mark on a 10 cm horizontal line with endpoints labelled “0-Extremely satisfied” and “10-Extremely dissatisfied” [48]. VAS dissatisfaction scores were calculated directly by measuring the distance of the line to the right of 0 [48]. Ratings were collected at different times during the experimental procedure; at the beginning of the experimental procedure (baseline level), at the end of the RHI without priming, and at the end of the RHI with priming (Fig. 1).

### Procedures

#### RHI method with or without distortion

The Rubber Hand Illusion procedure (RHI) is an experimental paradigm phenomenon where a synchronous touch of a real hand and an artificial hand results in the artificial hand being perceived as part of one’s own body [31]. In summary, the RHI is a somatosensory paradigm that can be produced when the participant sees a real-looking rubber hand being brushed with a paintbrush, while their real hand is hidden from view and brushed in a similar way with another paintbrush [31, 49]. Thus, stroking both hands (the real hand and the rubber hand) at the same time creates a sensory conflict between what you see and what you feel [31], and the procedure assesses how the brain resolves this discrepancy by adjusting the body representation to embody the fake hand [31]. Therefore, two paintbrushes [50] were used for stroking both the participant’s right hand and the rubber hand without distortion appearance (real-looking rubber) in a synchronous way for more embodiment [51]. It must be taken into account that during the illusion, aesthetic features of the rubber hand are incorporated into the mental representation of the body [52]. Therefore, to induce a rubber hand experience with the distortion effect (an incongruent visual image of the rubber hand), we used a very aged-looking rubber hand, i.e., a deteriorated rubber hand that is perceived with high dissatisfaction by the VAS (i.e. rubber hand procedure with distortion). We did not use a thin or heavy rubber hand to mask any possible body size bias. Both the BN-AN group and the healthy women were randomly assigned to one of two RHI conditions (Fig. 1). Thus, one condition was performed with a distorting rubber hand (BN-AN group n = 17 and HC group n = 13), whereas the other RHI was performed with a standard rubber hand (BN-AN group n = 16 and HC group n = 14). Immediately after each procedure, an ad hoc questionnaire [31] was administered to assess the following dimensions: (i) the location and proprioceptive drift, and (ii) the agency or motor control over the rubber hand [31]. Responses were measured on a 5-point Likert scale (1—total disagreement, 5—total agreement).

#### Priming effect experiment

To induce an experimental priming effect by exposure to thin media images, we have generated an adapted experimental priming protocol following Markis and McLennan’s [9]. The negative effect of the thin ideal appears to be greater when compared to the result of images of heavier bodies or neutral images [37]. Namely, we present a set of 25 images of thin body images, pre-selected by external researchers who rated them as highly representative of stereotypically slim female models wearing only underwear [10]. For the priming task, a Microsoft PowerPoint 2016 presentation was used to display the images [10]. Each image was presented for 10 s, with a 3-s interval between images, and the following participants rated every image [10]. For example, participants were asked to rate the extent to which each model matched their perception of the ideal female body as portrayed in the media, using a scale from 1 (“not at all”) to 4 (“very much”) [10]. These guidelines masked the fact that the purpose of viewing the photos was to expose participants to body dissatisfaction through social comparison [10, 37]. The priming task lasted 10 min in duration (Fig. 1). The RHI procedure was then performed, followed immediately by the questionnaire (Fig. 1).

### Data analysis

The present study can be considered a pilot study [53–55]. We determined our minimum sample size [56] using the IMIM application [57], which calculates our minimum sample size for comparing group means. For a total sample size of 33 patients and 27 healthy subjects, a minimum of 12 were required in each BN-AN subgroup (RHI with distorted appearance or RHI without distortion) and 6 in each HC subgroup (95% CI). SPSS^®^ v. 28 (SPSS, Chicago, IL) was used for all statistical analyses. The normality of the distribution was tested using the Shapiro–Wilk test, and the two-tailed t-test for the unpaired-sample or t-student for paired samples, or the non-parametric ones were U Man-Whitney/Wilcoxon, were used to calculate the statistical significance of differences in means between the two study groups or in the same group between subgroups. Alpha values of 0.05 and 0.01 were used as statistical significance (indicated where appropriate). Primary data are presented as means and standard errors. Likert scale data from individual questionnaire items were treated as ordinal data, whereas subscale scores were treated as interval data. Furthermore, we performed separate repeated measures mixed ANOVA on HC and BN-AN with 5 dependent variables (participants’ dissatisfaction (whole body, own hand, and rubber hand) and RHI embodiment index (location-proprioceptive drift, and agency)) × 2 between subject-factor (main effect group as RHI without distortion vs. RHI with distortion) × 2 levels among within-subject independent variables (without priming vs. priming effect). The effect size was determined by partial eta-squared. This analysis allowed us to determine if there was an effect of the priming condition on self-reported illusion intensity and perceived dissatisfaction, and if this effect differed depending on pre-existing dissatisfaction with the rubber hand. When relevant, significant interactions were broken down using Bonferroni-corrected post-hoc tests. Mentioned dissatisfaction and RHI embodiment indexes were also tested to be associated with the rubber hand distortion effect in each HC and BN-AN population using a binary logistic regression model. Predictor variables were included in the multivariate models if p < 0.20 and the forward Wald method was performed. All analyses were carried out separately in HC and BN-AN populations. The exponent of the regression coefficient Beta (β) as Odds ratio (OR), its 95% confidence interval, and statistical significance (p) are shown. In addition, we have estimated the relationships between variables (two-tailed Pearson's correlation).

## Results

The total sample consisted of 60 women: 33 right-handed women with a formal diagnosis of bulimia nervosa or anorexia nervosa (BN-AN) were evaluated (average age 24.35 years, SEM 3.90), as well as 27 control healthy women (HC) (average age 19.86 years, SEM 1.30), see Table 1 for clinical and demographic information. All patients were undergoing psychotherapy and psychiatric treatment for at least six months before the start of the study, so the present results are derived from clinically controlled patients. According to the SCL-90 questionnaire the mean depression and anxiety scores for our BN-AN sample were 2.24 (0.96) and 1.58 (0.94) respectively (Table 2). In our ED sample, 89.28% of patients scored above the cut-off point for the Global Severity Index factor. Compared with the reference values used for the validation of the questionnaire for the SCL-90 survey, all parameters were statistically significantly higher in the EDs for all items (Table 2).

**Table 1:** Demographic and clinical characteristics of participants

Variable	BN-AN patients (N = 33)	Healthy Controls (N = 27)
Age (years)	24.35 (3.9)	19.86 (1.3)
Education (years)	10.2 (1)	12.56 (0.7)
Current BMI (kg/m^2^)	18.30 (1.03)	–
Age at onset (years)	16.5 (2.36)	–

*BN-AN* bulimia nervosa or anorexia nervosa, *BMI* body mass index.

**Table 2:** BN-AN subjects characteristics according to the SCL-90 questionnaire

	BN-AN (n = 33)	Reference values SCL-90
SCL-90 R	M (SD)	M (SD)
Somatization	1.57 (0.87)	0.70 (0.61)
Obsessive–compulsive	2.20 (1.06)	0.63 (0.55)
Interpersonal sensibility	2.13 (0.97)*	0.48 (0.47)
Depression:	2.24 (0.96)**	0.84 (0.61)
Anxiety:	1.58 (0.94)	0.59(0.54)
Anger-hostility	1.22 (0.927)	0.48 (0.58)
Phobic-anxiety	1.01 (1.09)**	0.30 (0.42)
Paranoid ideation	1.57 (1.10)*	0.46 (0.53)
Psychoticism	1.41 (0.92)*	0.22 (0.32)
Global Severity Index (GSI)	1.78 (0.87)*	0.57 (0.40)
Total Symptom Positive ( TSP)	62.36 (19.01)	27.4 (14.8)
Positive Symptomatic Distress Positive Index IMSP)	2.16 (0.57)	1.80 (0.40)

Normative data of the reference population used for the validation of the questionnaire

*p < 0.05 or **p < 0.01 on the Student’s *t*-test for independent samples. Data are presented as mean (SD)

### Relationship between body shape dissatisfaction and interoceptive awareness

Subjects in the BN-AN sample scored an average of 79.40 (SEM 3.04) on the BSQ general score, which was significantly higher than healthy women (49.91, SEM 2.67). Indeed, these high scores were obtained in all domains of the BSQ, see Table 3, indicating greater dissatisfaction and discomfort with the experience of body image compared to HC. In addition, the differences in the domain measures of body dissatisfaction, e.g. “Concern about the unsightly aspects of obesity or general body dissatisfaction” or “Weight concern in connection with eating” were statistically highly significant, data summarised in Table 3. On the other hand, we used the BPQ to assess awareness of bodily processes. The global score of the Body Perception Questionnaire was higher in the BN-AN sample (2.74, SEM 0.14 vs. 2.36, SEM 0.11; p < 0.05). Furthermore, we found higher scores in the BN-AN sample on all subscales addressed, with statistically significant differences in most of them, see in Table 3. In addition, we found a strong correlation (two-tailed Pearson’s correlation r = 0.65, p < 0.001) between the intensity of the general score reported on the BSQ and the global score of the BPQ in the BN-AN sample, but not in the HC (r = 0.32, p = 0.092). The results show that the greater the body image dissatisfaction, the greater the awareness of one's body processes in BN-AN patients, and this positive association, consistent with increased body schema plasticity, can be interpreted as a reciprocal modulation.

**Table 3:** Ongoing body shape dissatisfaction and interoceptive awareness scores

	Healthy controls	BN-AN patients	
Body Shape Questionnaire (BSQ) domains			
Weight concern in connection with eating	18.34 (1.30)	31.96 (1.46)**	
Concern about the unsightly aspects of obesity	10.65 (0.94)	16.44 (0.93)**	
Concern about general body dissatisfaction	5.30 (0.44)	12.33 (0.66)**	
Body dissatisfaction regarding the lower body	5.78 (0.36)	9.44 (0.48)**	
Use of vomiting or laxatives to reduce body dissatisfaction	2.00 (0.00)	4.92 (0.49)**	
Body Perception Questionnaire (BPQ) domains			
Awareness	2.56 (0.17)		2.85 (0.17)
Stress response	2.79 (0.11)		3.26 (0.11)**
Autonomic nervous system reactivity	1.86 (0.15)		2.26 (0.10)*
Stress style 1	2.80 (0.18)		3.23 (0.21)*
Stress style 2	1.82 (0.11)		2.48 (0.11)*

Data from BN-AN patients and healthy controls on the Body Shape Questionnaire and the Body Perception Questionnaire

Scores of BN-AN patients were higher on all BSQ and BPQ subscales and statistically different on most subscales (*p < 0.05 or **p < 0.01 on the Student’s t-test for independent samples). Data are presented as mean (SEM)

### Reduction in perceived body dissatisfaction after RHI with distortion experience in BN-AN patients

#### Body dissatisfaction at baseline (before RHI experience; data recorded at t0)

**Fig. 2:**
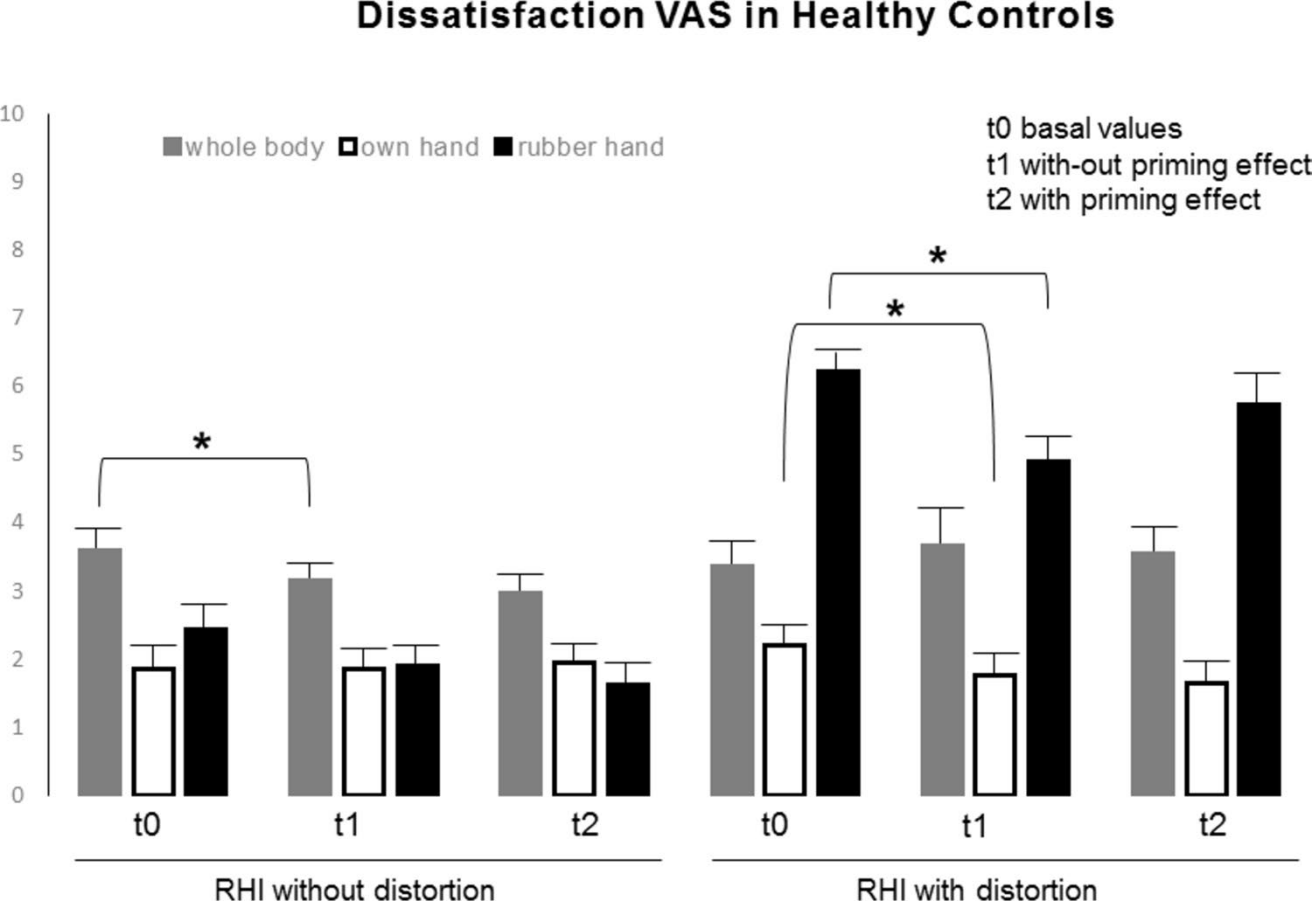
Ongoing dissatisfaction intensity scores of healthy controls at baseline and during the rubber hand illusion procedures. Subjects showed slightly higher levels of dissatisfaction intensity on the whole body and significantly higher levels on the distorting rubber hand during the RHI with distortion procedure (p < 0.05). Data are presented as mean and SEM

**Fig. 3:**
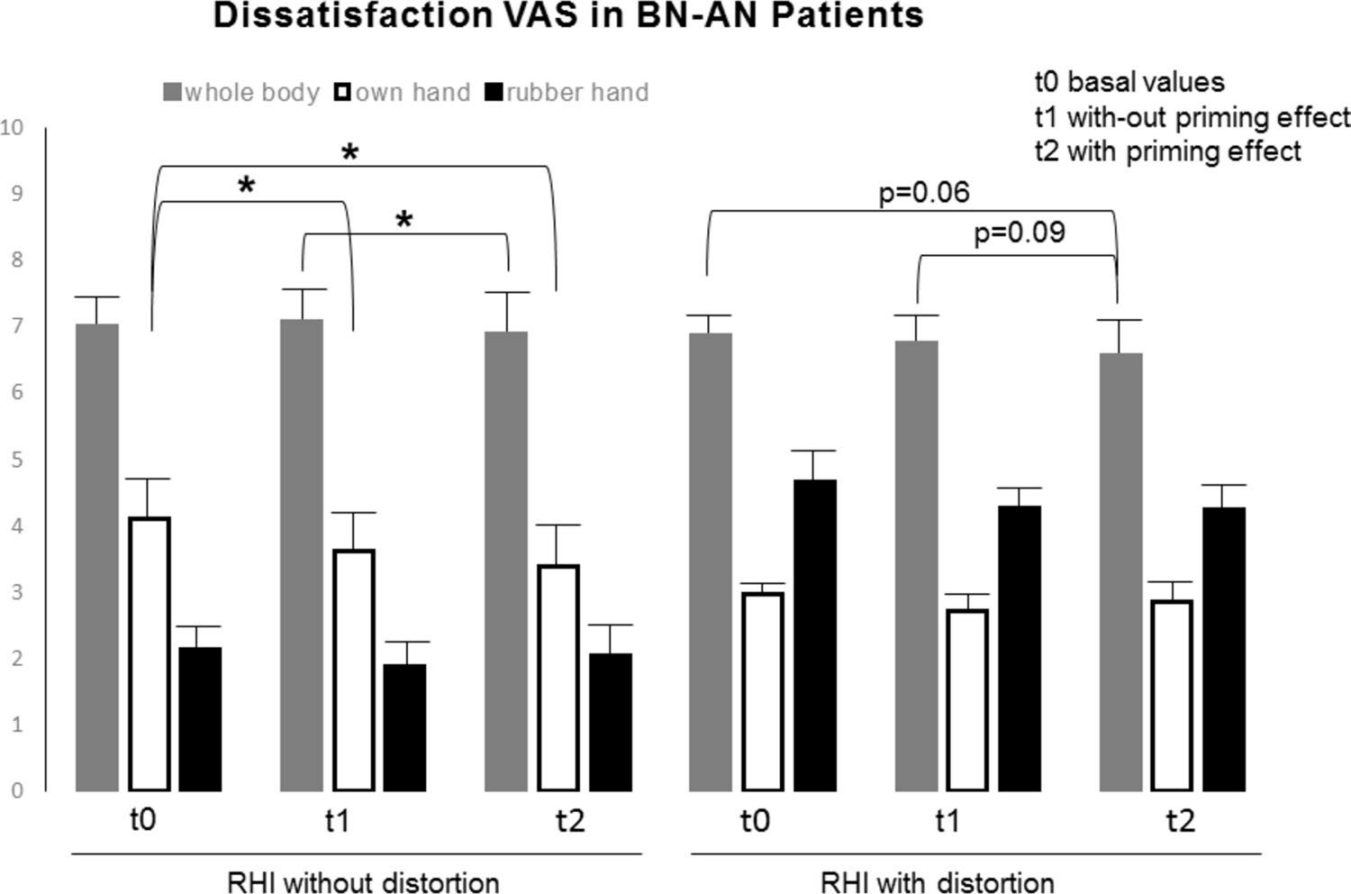
Ongoing dissatisfaction intensity scores of eating disorder patients at baseline and during the rubber hand illusion procedures. Subjects in the BN-AN sample exhibited significantly higher levels of body dissatisfaction intensity on the VAS, but these were lower on the RHI with the distortion procedure or priming effect. Data are presented as mean and SEM

The baseline results showed significant differences in perceived body dissatisfaction between the BN-AN group and healthy participants (Figs. 2, 3). Baseline VAS scores for the intensity of ongoing dissatisfaction reflected low levels of body dissatisfaction in the HC subjects (Fig. 2) and very high levels in the BN-AN sample (Fig. 3). In addition, high levels of dissatisfaction with the distorting rubber hand (relative to the standard rubber hand) were obtained in all experimental groups, although interestingly the values were higher in the HC sample (6.23, SEM 0.48 in HC vs. 4.66, SEM 0.83 obtained in BN-AN patients; Figs. 2 and 3). It is important to note that in the two BN-AN subgroups, the basal body dissatisfaction score was higher than the perceived one on a specific body part (e.g., 6.88–7.00, SEM 0.61–0.66 basal body scores vs. 2.97–4.01, SEM 0.54–0.89 own hand basal scores; Fig. 3). The data show that the perception of global body dissatisfaction is not the same as the perception of body parts or sections in ED patients (Fig. 3).

#### Body dissatisfaction after RHI

Contrast before the priming effect (t1 vs. t0) showed a slight reduction in body dissatisfaction in HC (3.59, SEM 0.45 vs. 3.15, SEM 0.53; p < 0.05; Fig. 2), but there were no significant changes due to the priming effect beyond the slight reduction in perceived dissatisfaction with the rubber hand (Fig. 2). However, during the experimental timing, perceived dissatisfaction with one's hand improved by 7.2% in the BN-AN patients (t1 vs. t0 and t2 vs. t0 p < 0.05; Fig. 3). In addition, no significant changes were registered in the body (7.00, SEM 0.66 vs. 7.08, SEM 0.76, t1 vs. t0; Fig. 3), but curiously after exposure to priming perceived body dissatisfaction decreased significantly by 1.9% (7.08, SEM 0.76 vs. 6.89, SEM 0.80, t2 vs. t1 p < 0.05; Fig. 3).

The data show that under control conditions (no distorting visual stimulus), the RHI experience per se slightly improves overall body dissatisfaction in HC but not in BN-AN subjects. In contrast, in the ED sample, the specific body part improves (e.g., the hand) but not the overall dissatisfaction; a distorting visual element is required to change the overall level in BN-AN patients.

#### Body dissatisfaction after RHI with distortion appearance

In the HC sample, there was no change in perceived body dissatisfaction, but there was a 13.3% decrease in dissatisfaction with the rubber hand and a 4.4% decrease in dissatisfaction with the own hand (t1 vs. t0, p < 0.05; Fig. 2). However, the priming effect slightly increased dissatisfaction with the rubber hand by 8.3% (t2 vs. t1; Fig. 2). The data obtained from the HC show that the distorting visual stimulus affects the dissatisfaction of a specific body part, but the whole body is not affected.

In contrast, in BN-AN patients, body dissatisfaction decreased by 1.2% before the priming effect (6.88, SEM 0.61 vs. 6.76, SEM 0.67, t1 vs. t0; Fig. 3) and continued to decrease by 1.9% after the priming effect (6.76, SEM 0.67 vs. 6.57, SEM 0.72, t2 vs. t1 p = 0.09; Fig. 3). In contrast, the intensity of dissatisfaction with either the own hand or the rubber hand did not change (Fig. 3). In short, in ED patients, visual stimulation of body images (either distorting or priming exposure) produces changes in body dissatisfaction after the RHI experience. That is, body perception through RHI with a visual distorting element of body images improves perceived body dissatisfaction.

### Body representation after the rubber hand illusion: location-proprioceptive drift embodiment plasticity in BN-AN patients

The differences between the two populations did not reach statistical significance, but BN-AN patients scored higher than HC subjects on all items of location-proprioceptive drift and agency domains when the experience was performed with standard RHI (at baseline condition), and these tendencies remained after the priming effect; see Tables 4 and 5. Moreover, in the BN-AN sample, prior to priming the differences in scores between the standard RHI and the distorted RHI were statistically different in location and proprioceptive drift domain (see Table 4). Namely, significantly lower scores were found for all items presented during the RHI with distortion experience, but these scores increased slightly after the priming effect (e.g., for the statement "It seemed as if the rubber hand was drifting toward my own hand" or "I felt as if my own hand was drifting toward the rubber hand"; Tables 4 and 5). In parallel, almost all items in the agency domain scored significantly lower during the RHI with distortion and increased after the priming effect (e.g., "It seemed as if I was unable to move my own hand" or "It seemed as if I could not really tell where my hand was"; Tables 4 and 5).

**Table 4:** Assessment of the RHI before priming effect in eating disorders and healthy controls

Controls	Healthy		Eating disorder (BN-AN)	
	Without distortion	With distortion	Without distortion	With distortion
Location and proprioceptive drift				
It seemed like the rubber hand was in the location where my hand was	4.27 (0.27)	3.38 (0.44)	4.12 (0.27)	3.70 (0.30)
I felt as if my own hand was drifting towards the rubber hand	2.63 (0.38)	2.07 (0.43)	3.56 (0.31)	1.82 (0.29)**
It seemed as if the touch I was feeling came from somewhere between my own hand and the rubber hand	2.63 (0.30)	2.69 (0.36)	3.87 (0.28)	2.35 (0.32)**
It seemed (visually) as if the rubber hand was drifting towards my own hand	2.54 (0.41)	2.07 (0.39)	3.18 (0.36)	1.76 (0.30)*
It seemed as if the rubber hand and my own hand were approaching	2.36 (0.36)	2.00 (0.35)	3.25 (0.37)	2.00 (0.32)*
Location and Proprioceptive Drift Index	2.88 (0.30)	2.44 (0.30)	3.59 (0.17)	2.32 (0.35)*
Agency				
It seemed like I was unable to move my own hand	2.81 (0.40)	3.07 (0.39)	3.75 (0.30)	2.23 (0.34)**
It seemed like I could not really tell where my hand was	3.45 (0.38)	3.23 (0.42)	3.31 (0.36)	2.41 (0.29)
It seemed like my own hand had disappeared	3.27 (0.42)	3.00 (0.37)	3.31 (0.32)	2.64 (0.29)
It seemed like my own hand was out of my control	2.90 (0.47)	3.00 (0.43)	3.25 (0.33)	2.47 (0.33)
It seemed like I could move the rubber hand if I would like	3.81 (0.32)	2.53 (0.40)	3.18 (0.33)	3.17 (0.35)
It seemed like I was in control of the rubber hand	3.81 (0.29)	2.53 (0.40)	3.18 (0.34)	3.00 (0.32)
Agency Index	3.34 (0.13)	2.89 (0.13)	3.33 (0.08)	2.65 (0.14)**
Global Index	3.57 (0.21)	2.85 (0.30)	3.67 (0.17)	2.93 (0.22)*

BN-AN patients’ scores on the proprioceptive drift and agency dimensions before the priming effect

Data from 5-point Likert scales (1—totally disagree, 5—totally agree) are presented as means (SEM; *p < 0.05, **p < 0.01 on the Mann–Whitney-Wilcoxon U-test between subgroups of the same population)

**Table 5:** Assessment of the RHI after the priming effect in eating disorders and healthy controls

Controls	Healthy		Eating disorder (BN-AN)	
	Without distortion	With distortion	Without distortion	With distortion
Location and proprioceptive drift				
It seemed like the rubber hand was in the location where my hand was	4.16 (0.27)	3.38 (0.47)	4.06 (0.35)	3.58 (0.35)
I felt as if my own hand was drifting towards the rubber hand	2.66 (0.28)	2.30 (0.41)	3.50 (0.40)	2.52 (0.34)
It seemed as if the touch I was feeling came from somewhere between my own hand and the rubber hand	2.58 (0.43)	2.61 (0.46)	3.50 (0.35)	2.35 (0.25)
It seemed (visually) as if the rubber hand was drifting towards my own hand	2.25 (0.27)	1.84 (0.31)	2.87 (0.37)	2.64 (0.36)
It seemed as if the rubber hand and my own hand were approaching	2.25 (0.32)	2.46 (0.40)	3.00 (0.41)	2.41 (0.35)
Location and Proprioceptive Drift Index	2.78 (0.30)	2.51 (0.30)	3.38 (0.21)	2.70 (0.22)
Agency				
It seemed like I was unable to move my own hand	3.08 (0.35)	3.07 (0.43)	3.43 (0.36)	2.41 (0.38)
It seemed like I could not really tell where my hand was	3.25 (032)	3.30 (0.41)	3.56 (0.41)	2.88 (0.34)
It seemed like my own hand had disappeared	3.66 (0.33)	3.23 (0.37)	3.68 (0.32)	2.76 (0.31)
It seemed like my own hand was out of my control	3.00 (0.30)	3.15 (0.46)	3.56 (0.37)	2.76 (0.32)
It seemed like I could move the rubber hand if I would like	3.66 (0.35)	2.53 (0.35)	3.37 (0.40)	3.05 (0.36)
It seemed like I was in control of the rubber hand	3.66 (0.33)	2.92 (0.44)	3.50 (0.40)	3.17 (0.37)
Agency Index	3.38 (0.13)	3.03 (0.13)	3.51 (0.04)	2.83 (0.10)**
Global Index	3.59 (0.12)	3.02 (0.31)	3.63 (0.27)	3.07 (0.24)

BN-AN patients’ scores on proprioceptive drift or agency dimensions after the priming effect

Data from 5-point Likert scales (1—totally disagree, 5—totally agree) are presented as means (SEM; **p < 0.01 on the Mann–Whitney-Wilcoxon U-test between subgroups of the same population).

In light of the documented change in body dissatisfaction during the RHI with distortion and plasticity in the location-proprioceptive domain, we sought to investigate the relationship between these variables. In order to achieve this, we employed mixed repeated measures (ANOVA) and regression analysis. We computed repeated measures ANOVAs for each of the subgroup measures before and after the priming effect, and interestingly, only the RHI with distortion BN-AN subgroup and before the priming effect showed an interaction (F = 4.426 (df = 1), p = 0.052, η2 = 0.217) in contrast with the RHI standard condition (F = 0.964 (df = 1), p = 0.342, η2 = 0.060). Thus, the RHI with a distortion appearance was effective in disrupting the location-proprioceptive drift embodiment index, and this disruption was associated with an improvement in body dissatisfaction in BN-AN patients. In addition, before the priming effect, the two main univariate associations remained significant in the regression, indicating that they were loosened during exposure to thin body images. I.e., the index of location-proprioceptive drift, which was negative (the embodiment index decreased to a level similar to HC (OR = 0.231; 95% CI [0.087–0.612]; p = 0.003) and the decrease in body dissatisfaction (OR = 0.907; 95% CI [0.841–0.978]; p = 0.012).

## Discussion

The current study recorded evidence of proprioceptive distortion and high interoceptive awareness in patients with bulimia nervosa or anorexia nervosa, which is consistent with the presence of a maladaptive body schema. Furthermore, the influence of visual exposure to distorting aesthetic or thin-body media images on in situ body representation was analysed, with a focus on the proprioceptive and agency embodiment domains. Our data indicate, for the first time in BN-AN patients, that visual body image incongruence is associated with changes in proprioceptive embodiment and reduced body dissatisfaction. However, cognitive bias due to exposure to thin-body images leads to a maladaptive body schema.

The body schema is constantly recalibrated by perceptual information from multisensory sources, and embodiment effects are known to occur during the rubber hand illusion [58]. It is well established that individuals with eating disorders are more susceptible to the rubber hand illusion than healthy controls [34, 52, 59]. However, the precise mechanisms by which maladaptive body schemas are recalibrated by new somatosensory input in patients with bulimia or anorexia nervosa remain unclear. In this context, the present study reports that a number of factors may be associated with a maladaptive body schema in patients with BN or AN. Firstly, the proprioceptive recalibration after new somatosensory input depends on the visualized body image. In the presented data, the aesthetic features that were perceived as unsatisfactory but with a normal body size (neither fat nor thin) were pivotal in the phenomenon of proprioceptive embodiment. It is well stablished, that body-related representations are derived from both on-line afferent somatosensory inputs and off-line representations stored in long-term memory [24, 60]. Also, it is known that EDs may be the result from an impairment in the ability to update a negative body representation stored in autobiographical memory with real-time sensorimotor and proprioceptive data [61, 62], which may be influenced by visual perception and cognitive flexibility deficits [61]. Moreover, in this experimental context, the body representation derived from on-line somatosensory input is influenced by viewing an incongruent body image, and resulting in a reduction in body dissatisfaction. One possible interpretation is that the maladaptive body schema is being modulated by body image in BN-AN patients. This is evidenced by the observation that the embodiment of proprioception decreases at scores similar to those observed in healthy controls. Furthermore, when visual body image influence is associated with the preservation of proprioception, there is an improvement in body dissatisfaction.

From a neuroanatomical perspective, recent data suggest a correlation between the embodiment of proprioception by rubber hand illusion and body representation, which are processed in the same brain area, namely the extrastriate body area (EBA) [63–65]. Indeed, neuroimaging studies have shown a correlation between atypical visual processing and body misperception within the EBA, which is associated with body image disturbances in women with ED [66], and this evidence suggests changes in functional and structural activity [67–70]. The EBA is also known to be involved in the perceptual component of body image disorders, which involves the aesthetic evaluation of body stimuli [71–75]. In addition, visual attention to presented stimuli (such as body images) has been associated with increased activity in the EBA in women with binge eating disorder [66] and it has been postulated that this may activate (or prime) an individual’s maladaptive body schema through social comparison and self-discrepancy [37]. In this context, our findings align with this hypothesis, as the impact of exposure to thin-body images on body schema was observed, with some recovery of proprioceptive distortion, which is consistent with the influence of one's body image on distorted body representation. In other words, cognitive biases contribute to the maladaptive body schema, which may be more or less dependent on the nature of the effect of visual body images. It appears that an unsatisfactory image may act as a block to online proprioceptive distortion, whereas exposure to thin-body media images may promote distortion in BN-AN patients.

The second characteristic of the maladaptive body schema, as evidenced by the present data, is that the greater the body dissatisfaction, the greater the interoceptive awareness in patients with bulimia or anorexia nervosa. Recent studies have indicated that ED pathology may be related to a core impairment in interoception, or the ability to perceive and modulate the physiological condition of the body [76–79]. Additionally, a discrepancy between reported and actual interoceptive states has been found in people who have recovered from AN [80]. In this regard, data from our BN-AN sample indicated higher scores on the BPQ, which assesses awareness of one's bodily processes. Furthermore, correlational analyses showed that greater body shape dissatisfaction, as measured by the BSQ, was associated with greater awareness of bodily processes. Analogous to the findings in individuals suffering from chronic pain [31], a potential interpretation of the modulation of body dissatisfaction and body awareness is that heightened body awareness would increase body dissatisfaction, i.e., a state of hypervigilance toward internal sensations would contribute to dissatisfaction and body image disturbance. Recent studies have shown that enhanced cortical representation of interoceptive stimuli enhances the perception of negative physiological states associated with food intake, thereby promoting fasting as a means of alleviating negative bodily states [80] and that heightened sensitivity to somatic concerns may, in turn, perpetuate ED symptoms and mutually reinforce other somatic concerns [81, 82]. It can be postulated that body image disturbance in patients with bulimia or anorexia nervosa may result in the activation of a state of hypervigilance towards interoceptive awareness, which would act as an underlying mechanism of the maladaptive body schema. In light of these findings, it is evidence that the clinical importance of modulating the body schema and regulating the imbalance between proprioception and interoception to improve body dissatisfaction in patients with bulimia or anorexia nervosa should be considered.

There are several methodological limitations that should be taken into account. Firstly, the BSQ and/or BPQ questionnaires were not administered at the end of the RHI experimental procedure to assess changes in the registered proprioceptive change mediated by the visual stimulus. Secondly, it would be beneficial to extend studies utilizing this or analogous methodologies to investigate changes in response to alternative visual distortion stimuli (e.g., a very thin or thick rubber hand or a priming effect by body images of fat or elderly women).

It should be noted that this is a pilot study and the results should be considered as preliminary due to the small sample size considered. The results suggest that the exposure effect of thin-body media images may trigger the maladaptive body schema in patients with bulimia or anorexia nervosa. Conversely, visual body images that are perceived as unsatisfactory play a role in modulating the body schema, in particular in preserving proprioception and consequently in reducing body dissatisfaction. In conclusion, the maladaptive body schema in patients with bulimia or anorexia nervosa appears to be characterised by distorted proprioception and heightened interoceptive awareness, which directly correlate with high perceived dissatisfaction.

## Data Availability

Raw data that support the findings of this study are available from the corresponding author, upon reasonable request.
